# Speciation study of the heavy metals in commercially available recharge cards coatings in Nigeria and the health implication

**DOI:** 10.1016/j.toxrep.2014.05.008

**Published:** 2014-05-22

**Authors:** Abolanle S. Adekunle, John A.O. Oyekunle, Suliat O. Baruwa, Aderemi O. Ogunfowokan, Eno E. Ebenso

**Affiliations:** a Department of Chemistry, Obafemi Awolowo University, Ile-Ife, Nigeria; b Department of Chemistry, School of Mathematical and Physical Sciences, North-West University (Mafikeng Campus), Private Bag X2046, Mmabatho 2735, South Africa

**Keywords:** Heavy metals, Speciation, Mobile phones, Recharge cards, Health effect, XRF analysis

## Abstract

This work assessed levels of heavy metals exposure from silver coatings of mobile phones recharge cards of three major companies (designated as A, B and C) with price denominations  100,  200 and  400 from companies A, B and C respectively, which were carefully scratched using a plastic scraper into a glass tube. The coatings were acid digested for total metal concentration, while speciation experiment for Mn, Cu, Cd and Pb was carried out. Total metals and speciation analysis were done using AAS and XRF techniques. The total metal concentration from XRF analysis was in the range: Ca (70–2140 μg/g), K (20–4930 μg/g), Sc (80–270 μg/g), Ti (1530–12,580 μg/g), Fe (50–6660 μg/g), Ni (20–2040 μg/g), Cu (20–850 μg/g) and Zn (40–460 μg/g). Cr had the lowest concentration (10 μg/g) in A ( 400) while Ti had the highest concentration (12,580 μg/g) in C ( 500) for all the coatings analyzed. AAS and XRF results agreed closely except for Fe with higher concentration. A ( 100) contained high concentration of the metals compared with others. Speciation study identified Mn as the most mobile element when present in the environment.

## 1 Introduction

Heavy metals have been shown to abound in several environmental matrices including water [18], plants [17], soil [24], air particulates [12], cosmetics [15] and even biological tissues and organs [33] to mention a few. Some of these heavy metals are essential elements for human growth and development, but could pose serious health problems at very high concentration in the body. For example, zinc (Zn) is important for the physiological functions of living tissue and regulates many biochemical processes but too much Zn can cause imminent health problems, such as stomach cramps, skin irritations, vomiting, nausea and anemia [25]. Copper (Cu) does essential work in animal metabolism but excessive ingestion of copper brings about serious toxicological concerns, such as vomiting, cramps, convulsions, or even death [27]. Nickel (Ni) exceeding its critical level might bring about serious lung and kidney problems aside from gastrointestinal distress, pulmonary fibrosis and skin dermatitis; Ni is also known as a human carcinogen [2]. High concentrations of mercury (Hg) cause impairment of pulmonary and kidney function, chest pain and dyspnea [20], while chronic exposure of cadmium (Cd) results in kidney dysfunction and high levels of exposure will result in death. Lead (Pb) can damage the kidney, liver, reproductive system, basic cellular processes and brain functions. The toxic symptoms are anemia, insomnia, headache, dizziness, and irritability, weakness of muscles, hallucination and renal damages.

Although several adverse health effects of heavy metals have been known for a long time, the exposure to these elements continues and is even increasing in the less developed countries where several people still remain ignorant of how to curb the menace of heavy metal exposure. One of the present day exposure routes to heavy metals is through the use of mobile phone recharge cards which are becoming increasingly popular among the young and the old [23]. Hence, the associated health risk of the heavy metal content of these recharged cards based on their species motivated the present study. To the best of our knowledge, the present speciation study represents the first of its kind with respect to recharge cards usage.

Chemical speciation of heavy metals describes their composition, forms of association, ionic types and concentration in a given matrix. The significance of chemical speciation for the environmental sciences lies in its usefulness as a tool for the interpretation of chemodynamics, properties and toxicity of chemical compounds. Chemical speciation depends, amongst other factors, on the composition of the system, pH, temperature, ionic strength and time. Many studies dealing with particulate metals in natural water systems (i.e. metal associated with suspended matter or bottom sediments) including roadside deposits are usually concerned with total metal concentration [26], [14], [16], [19], [13]. Relatively few attempts have been made to evaluate the speciation of particulate metals in sub-urban center particularly among the various forms in which they might exist even in other matrices including recharge cards silver coatings.

Use of total metal concentration as a criterion to assess their potential effects in the environment is important but it implies that all terms of a given metal have an equal impact on the environment; such an assumption is clearly unacceptable. However, a comprehensive knowledge of interaction between the different species of the metal and environmental media is important in predicting their environmental impacts and consequent health risk.

Since the advent of recharge cards in the world especially for commercial consumption by the regular cell phone users, little or very scarce reports on its heavy metals speciation have been reported. For example, Okunola et al. [23] reported the presence of total iron, nickel, chromium, manganese, copper, zinc, lead, and cadmium in commercially available recharge cards but not the species of the metals. Assessing the health risk associated with this metals is important since it is a common habit among people using recharged cards especially in Nigeria to use their finger nails to scratch the recharge card coating without proper washing of the nails thereby contributing a direct dermal contact with the heavy metal content of the coating films. Also in the course of removing the coating or scratching, the scratched particles are carelessly dispersed in the environment. Some of the particles contaminate the air around, while in most cases, larger percentage are returned into the ground where they are subjected to a number of processes that influences their mobility in the soil as well as their potential to contaminate ground water. Further transformations may lead to contamination of farm products and fresh water plants and animals, and finally get into the food chain. Thus, the human health implication of these heavy metals in the recharge card coatings, either in their total, free or bonded form cannot be over emphasized.

This study was aimed at determining the levels of heavy metals and their species in silver coating films of commercially available recharged cards in Nigeria and assessing the health implications of such practices on humans. It also attempted to evaluate their long term stability tendency when present in the environment.

## 2 Materials and methods

### 2.1 Sample collection and preparation

Mobile phone recharge cards from three major recharge cards producing companies in Nigeria were used for this study. The companies are denoted as A, B and C, respectively. The recharge cards commonly and commercially available are in the price denominations of  100,  200,  400,  500,  750, and  1500, respectively, with the  100 denomination most purchased by a greater percentage of the population. More than hundred (100) samples of each denomination were obtained from the recharge cards sellers and retail shops in Ile-Ife, Osun-State, Nigeria. The denominations obtained and studied for each company were: A ( 100,  200,  400); B ( 200,  500); and C ( 200,  500), respectively. The silver coatings were carefully scratched off using a plastic scraper into a glass tube. Adequate care was taken during sample collection to avoid cross contamination. The samples were later analyzed for their metal composition using both X-ray fluorescence (XRF) and Atomic Absorption Spectroscopic (AAS) techniques.

### 2.2 Reagents used and treatment of containers

Reagents used were of analytical grade (Sigma, Merck and BDH chemicals) and include ammonium acetate (NH_4_COOCH_3_), sodium acetate (CH_3_COONa), hydroxylamine hydrochloride (NH_2_OH·HCl), acetic acid (CH_3_COOH), nitric acid (HNO_3_) and hydrogen peroxide (H_2_O_2_). Solutions were prepared using doubly distilled-deionised water. All glassware used were washed in detergent solution, rinsed several times with distilled-deionised water and then soaked for 48 h in 10% HNO_3_, after which they were rinsed further with distilled-deionised water and dried overnight in an oven at a temperature of 120 °C before used [37]. Plastic bottles (60 mL) needed for AAS sample preparation were cleaned using (1:1) HCl–water solution. The syrup bottles were fully filled with the 1:1 HCl solution and left for 24 h, after which they were rinsed thoroughly with distilled-deionised water and dried.

### 2.3 Total metal determination (XRF analysis)

In order to acquire the spectrum in the X-ray machine, 1.0 g of the silver coating of recharge cards of different dominations were made into a pellet in a Spec-Cap 13 mm diameter with the aid of hydraulic press, while aluminum foil was used to hold them in place. The pelletized sample was irradiated for 1000 s to acquire X-ray fluorescent peaks using X-ray fluorescence spectrophotometer (VGP Model 201, USA) available at the Center for Energy Research and Development, Obafemi Awolowo University, Ile-Ife, Nigeria. The respective elements (Cd, Pb, Cr, Cu, Ni, Fe, Zn etc.) were identified from their corresponding peaks and their concentrations determined. The result was analyzed using software (XRF-FP Quantitative Analysis Software).

### 2.4 Total metals determination (AAS analysis)

Accurately weighed sample (0.025 ± 0.001 g) of the silver coatings sample was placed in a digestion tube and digested with a mixture of HNO_3_ and HClO_4_ (4:1 ratio) for metal determination using the procedure described by Okunola et al. [23]. The sample solutions were analyzed for concentration of Cd, Pb, Cr, Cu, Ni, Fe and Zn in an air-acetylene flame using Flame Atomic Absorption Spectrophotometer (Buck Scientific AAS 205 Model, USA). The equipment was calibrated using working standards 0, 2, 4, 6, 8 and 10 ppm of each metal serially prepared from their 1000 ppm reference standards obtained from Fisher's AAS Inc., USA. Concentrations of the working ranges were obtained by diluting an appropriate volume of the stock solution with ultra pure water.

The equipment was previously standardized and corrected for background metal impurities using a blank determination. The samples were analyzed in triplicates.

### 2.5 Speciation study using sequential extraction method

The extraction protocol has been validated for the selectivity of various fractions of trace elements in metals, soil, sediment samples and dust [31]. The method involved weighing a known amount of sample (in the present study, 0.2 g) and subjecting it to the various extractant treatments for exchangeable ions (F1), carbonate-bound ions (F2), Mn-oxide bound ions (F3), organic fraction (F4) and residual fraction (F5) as described by Tessier et al. [31].

#### 2.5.1 Mobility factor

The relationship adopted by Ogunfowowokan et al. [22] was used to determine the mobility factor of the metals. This relationship is:

$$\text{MF}=\frac{\text{F}1+\text{F}2}{\text{F}1+\text{F}2+\text{F}3+\text{F}4}\times 100$$

### 2.6 Statistical analysis

Coefficient of variation (CV) is chosen as a statistical tool to explain the distribution patterns of the metals in the recharge cards. CV was calculated using the relationship:$\text{CV}=\frac{\text{s}.\text{d}.}{\bar{X}}\times 100$

where s.d., standard deviation,$\bar{X}$, mean.

## 3 Results and discussion

The XRF and the AAS results of the total metals concentration in different major silver coated recharge cards are presented in Table 1, Table 2 respectively, while the speciation results are presented in Table 3, Table 4, Table 5, Table 6. For the XRF, the elements detected were in the range: Cl (10–2300 μg/g), Ca (70–2140 μg/g), K (20–4930 μg/g), Sc (80–270 μg/g), Ti (1530–12,580 μg/g), Cr (10–440 μg/g), Fe (50–6660 μg/g), Ni (20–2040 μg/g), Cu (20–850 μg/g) and Zn (40–460 μg/g).

**Table 1:** Concentration (μg/g) of heavy metals in silver coatings of recharge cards obtained by XRF analysis.

Recharge cards denominations	Cl	Ca	Sc	Ti	Cr	Fe	Ni	Cu	Zn	K
A ( 100)	2300.000 ± 0.017	1760.000 ± 0.008	80.000 ± 0.001	1530.000 ± 0.005	440.000 ± 0.002	3630.000 ± 0.006	1030.000 ± 0.003	850.000 ± 0.003	410.000 ± 0.002	–
A ( 200)	250.000 ± 0.000	120.000 ± 0.001	190.000 ± 0.001	11,420.000 ± 0.001	30.000 ± 0.000	520.000 ± 0.001	150.000 ± 0.001	150.000 ± 0.000	60.000 ± 0.000	100.000 ± 0.001
A ( 400)	10.000 ± 0.000	80.000 ± 0.000	270.000 ± 0.001	12,310.000 ± 0.004	10.000 ± 0.000	120.000 ± 0.000	40.000 ± 0.000	40.000 ± 0.000	40.000 ± 0.000	20.000 ± 0.000
B ( 200)	1290.000 ± 0.009	650.000 ± 0.004	–	1540.000 ± 0.004	90.000 ± 0.001	6660.000 ± 0.007	1670.000 ± 0.004	310.000 ± 0.002	460.000 ± 0.002	340.000 ± 0.004
B ( 500)	–	2140.000 ± 0.008	140.000 ± 0.002	2490.000 ± 0.006	–	1020.000 ± 0.003	2040.000 ± 0.004	240.000 ± 0.001	–	4930.000 ± 0.015
C ( 200)	–	120.000 ± 0.000	270.000 ± 0.001	12,470.000 ± 0.005	–	80.000 ± 0.000	30.000 ± 0.000	20.000 ± 0.000	–	20.000 ± 0.000
C ( 500)	–	70.000 ± 0.000	240.000 ± 0.001	12,580.000 ± 0.005	–	50.000 ± 0.000	20.000 ± 0.000	20.000 ± 0.000	–	20.000 ± 0.000
Mean ± s.d.	550 ± 903.62	705.71 ± 880.68	170.00 ± 102.63	7762.86 ± 5549.61	81.43 ± 161.39	1725.71 ± 2517.27	711.43 ± 865.44	232.86 ± 294.83	138.57 ± 204.32	775.71 ± 1835.74
CV	164.29	124.79	60.37	71.50	198.19	145.87	121.65	126.61	147.45	236.65

**Table 2:** Mean concentrations (μg/g) of the heavy metals in silver coating of recharge cards obtained by AAS analysis.

Recharge cards denomination	Fe (μg/g)	Zn (μg/g)	Cu (μg/g)	Ni (μg/g)	Cd (μg/g)	Pb (μg/g)	Ag (μg/g)	Cr (μg/g)
A ( 100)	64,469.000 ± 1214.809	8354.000 ± 73.539	661.000 ± 18.385	61.000 ± 1.414	15.000 ± 1.414	21.000 ± 1.414	10,449.000 ± 74.953	7.000 ± 1.414
A ( 200)	42,070.000 ± 175.362	7634.000 ± 53.740	394.000 ± 2.828	51.000 ± 1.414	11.000 ± 1.414	24.000 ± 2.828	9769.000 ± 386.080	8.000 ± 0.000
A ( 400)	50,546.000 ± 212.132	9386.000 ± 141.421	644.000 ± 11.314	59.000 ± 1.414	15.000 ± 1.414	19.000 ± 7.071	12,108.000 ± 189.505	9.000 ± 1.414
B ( 200)	70,000.000 ± 0.000	9997.000 ± 49.497	617.000 ± 29.698	58.000 ± 2.828	16.000 ± 0.000	18.000 ± 0.000	10,483.000 ± 156.978	7.000 ± 1.414
B ( 500)	56,410.000 ± 186.676	6796.000 ± 217.789	555.000 ± 24.042	56.000 ± 2.828	11.000 ± 1.414	21.000 ± 1.414	9739.000 ± 388.909	7.000 ± 1.414
C ( 200)	40,018.000 ± 96.167	5996.000 ± 33.941	395.000 ± 29.698	63.000 ± 1.414	20.000 ± 2.828	18.000 ± 0.000	11,420.000 ± 53.740	7.000 ± 1.414
C ( 500)	47,526.000 ± 701.449	7587.000 ± 202.233	499.000 ± 4.243	57.000 ± 1.414	13.000 ± 1.414	15.000 ± 1.414	10,345.000 ± 196.576	10.000 ± 0.000
Mean ± s.d.	53,005.60 ± 11,226.08	7964.29 ± 1402.11	537.86 ± 112.25	57.86 ± 3.85	14.43 ± 3.15	19.43 ± 2.88	10,616.10 ± 863.91	7.86 ± 1.21
CV	21.18	17.60	20.87	6.65	21.83	14.82	8.14	15.39

**Table 3:** Manganese levels (μg/g) in the different fractions of the recharge card coatings.

Samples	Exchangeable	Carbonate bound	Mn-oxide bound	Organic bound	Residual	Total
A ( 100)	938.875	747.125	992.000	782.250	924.625	3641.875
A ( 200)	567.000	534.875	760.75	619.375	744.500	3226.500
A ( 400)	743.875	647.375	872.875	724.500	873.500	3862.125
B ( 200)	535.875	498.000	743.375	568.500	689.125	3034.875
B ( 500)	866.500	698.000	905.750	755.500	893.500	4119.250
C ( 200)	666.250	602.750	817.625	688.500	842.625	3617.750
C ( 500)	610.750	564.250	786.875	652.125	790.625	3404.625
Mean ± s.d.	704.16 ± 152.93	613.20 ± 89.63	839.89 ± 88.93	684.39 ± 76.33	822.64 ± 85.01	
CV	21.72	14.62	9.14	11.15	10.33

**Table 4:** Copper levels (μg/g) in the different fractions of the recharge card coatings.

Samples	Exchangeable	Carbonate bound	Mn-oxide bound	Organic bound	Residual	Total
A ( 100)	217.375	121.625	251.750	147.375	124.625	862.750
A ( 200)	79.625	82.375	186.125	94.875	71.750	514.750
A ( 400)	108.375	94.750	206.375	119.500	82.000	611.000
B ( 200)	76.750	74.625	92.000	89.125	69.000	311.500
B ( 500)	151.000	99.375	228.125	129.375	103.875	711.750
C ( 200)	101.375	87.125	134.750	101.625	80.875	505.750
C ( 500)	87.250	85.125	118.000	100.250	74.000	464.625
Mean ± s.d.	117.39 ± 50.75	92.14 ± 15.30	173.88 ± 59.96	111.73 ± 21.10	86.59 ± 20.36	
CV	43.23	16.61	34.48	18.88	23.51

**Table 5:** Cadmium levels (μg/g) in the different fractions of the recharge card coatings.

Samples	Exchangeable	Carbonate bound	Mn-oxide bound	Organic bound	Residual	Total
A ( 100)	2.625	2.375	4.250	2.750	2.375	14.375
A ( 200)	1.000	0.875	2.250	1.125	0.875	6.125
A ( 400)	2.125	1.750	3.500	2.125	1.625	11.125
B ( 200)	0.625	0.625	1.750	0.625	0.625	4.25
B ( 500)	2.375	2.125	3.875	2.625	1.875	12.875
C ( 200)	1.750	1.375	3.125	1.875	1.250	9.375
C ( 500)	1.125	1.125	2.625	1.375	1.000	7.250
Mean ± s.d.	1.66 ± 0.76	1.46 ± 0.65	3.05 ± 0.90	1.79 ± 0.79	1.38 ± 0.62	
CV	45.78	44.52	29.51	44.13	44.93

**Table 6:** Lead levels (μg/g) in the different fractions of the recharge cards coatings.

Samples	Exchangeable	Carbonate bound	Mn-oxide bound	Organic bound	Residual	Total
A ( 100)	3.375	3.125	4.875	3.125	2.625	17.125
A ( 200)	1.375	1.625	2.625	1.375	1.125	8.125
A ( 400)	2.625	2.750	3.875	2.625	2.125	14.000
B ( 200)	1.000	1.250	2.000	1.000	0.875	6.125
B ( 500)	3.125	2.875	4.250	2.875	2.375	15.500
C ( 200)	2.250	2.375	3.625	2.250	1.750	12.250
C ( 500)	1.875	2.125	3.125	1.750	1.375	10.250
Mean ± s.d.	2.23 ± 0.88	2.30 ± 0.68	3.48 ± 0.98	2.14 ± 0.80	1.75 ± 0.66	
CV	39.46	29.57	28.16	37.38	37.71	

Chromium had the lowest concentration of the heavy metals (10 μg/g) in A ( 400) while Ti had the highest concentration (12,580 μg/g) in C ( 500) for all the coatings analyzed. Results showed that A ( 100) which is the readily affordable denomination by most users of recharge cards contained very high concentrations of the heavy metals compared with other samples investigated, and therefore represent a higher health risk. The heavy metals concentration and distribution in A ( 400), along with C ( 200) and C ( 500) were lower and safer compared with others and are considered relatively safe for the buyers and sellers of the product.

Table 2 presents the results of the AAS analysis of the recharge cards silver coatings. The metals are in the range Fe (39950–70000 μg/g), Zn (5972–9962 μg/g), Cu (374–652 μg/g), Ni (52–62 μg/g), Cd (12–18 μg/g), Pb (16–26 μg/g), Ag (9464–12242 μg/g) and Cr (6–10 μg/g).

The results tend to reflect similar order of magnitude with the XRF results except for Fe and Zn with about 10–100 times the concentration from the XRF results. This may be due to the different methods of sample preparation, matrix effect and technique used. The results also suggested that AAS may be better method of analysis of the samples due to matrix effects. At present, there are very scarce or limited literatures on heavy metals in recharge cards silver coatings to compare results obtained in this study with. The only available literature by Okunola et al. [23] showed that the AAS results obtained in this study for Fe (39950–70000 μg/g) and Zn (5972–9962 μg/g) compared favorably in magnitude with high values of Fe (11,397–75,525 μg/g), and Zn (561–23,691 μg/g) reported for recharge cards coatings using similar technique [23]. AAS results also indicated that Cr, Cd and Pb occurred at relatively low concentrations in the silver coatings. However, the highest concentration of Cr (10 μg/g), Cd (18 μg/g) and Pb (26 μg/g) obtained in this study are lower compared with highest values of 7025.5, 222.5 and 1429.0 μg/g respectively reported for the metals in silver coatings [23]. Very high Ag concentration recorded in this work is not surprising as Ag itself is used as support for the electronic films coated on the recharge cards. Recently, its use in recharge cards coating has been discouraged due to the associated cancer related diseases [6].

The AAS results also followed a similar trend for the heavy metal concentration and distribution earlier observed in the different denominations under XRF results. For example, A ( 100), B ( 200) and B ( 500) denominations have high concentration of the heavy metals compared with C ( 200) and C ( 500). The sources of these heavy metals in the silver coating could be due to the use of their oxides as mordant or colorants for esthetic purposes during production. It may also be due to trace quantities of these heavy metals in the raw materials, or in the water used during production processes. Thus, from the XRF and the AAS results, it was observed that silver coatings of products from companies A and B had higher concentrations of these heavy metals, therefore serious measures to minimize and control all possible sources of the metals incursion into these products should be pursued.

Presently, there is no set standard either locally or internationally for heavy metal concentration in recharge cards coatings; it is therefore difficult to ascertain if the values obtained in this study especially from the AAS results are too high or too low. However, considering the allowable set standards of 0.1, 0.1, 50, 0.05, 1.0 and 1.0 mg/L for Pb, Mn, Zn, Cr, Fe and Cu respectively in drinking water [34]; and 70 mg kg^−1^ Pb, 1–10 mg kg^−1^ Cd, 200–600 mg kg^−1^ Zn [7], [35], [36] and 100 mg kg^−1^ Cu [30] in soil, we may adjudge the data obtained in this study as relatively high and alarming to human health for a product like recharge cards that is consumed daily by many cell or mobile phones users in Nigeria. This study has also demonstrated that mobile recharge cards coating could serve as sources of human exposure to heavy metals through dermal contact to human system. Therefore users are advised to be more careful in the use of the products. For example, studies have shown sign of cadmium induced kidney damage in the general population at urinary cadmium levels of 2–3 μg/g creatinine [3], [11]. In fact, Cd and Cr are prohibited in any amount in cosmetics. Since the heavy metals can get to the human system through dermal exposure by adhering of the silver coatings on nails, or under the fingers contaminating food we eat, therefore consumers should be very careful in the way they scratch and handle the mobile recharge cards. For example, dermal exposures to chromium have been associated with skin rashes, kidney and liver damage, lung cancer, respiratory problems and even death (http://www.lenntech.com/periodic-chart-elements/cr-en.htm). Copper manifests at low concentrations and exposure to copper compound dust can cause dermatitis, discoloring of the skin, irritation of the nose and throat, while chronic exposure can cause brain damage [32], [1]. Lead can cause central nervous system damage. Lead can also damage the kidney, liver and reproductive system, basic cellular processes and brain functions. The toxic symptoms are anemia, insomnia, headache, dizziness, irritability, weakness of muscles, hallucination and renal damages [21]. Nickel exceeding its critical level might bring about serious lung and kidney problems aside from gastrointestinal distress, pulmonary fibrosis and skin dermatitis [2]. Zinc can cause eminent health problems, such as stomach cramps, skin irritations, vomiting, nausea and anemia [25]. Therefore, the use and incorporation of heavy metals in recharge cards coating calls for immediate regulation and control by the appropriate authority in order to check their accumulated health effects over a prolong usage.

To further appraise the health risk associated with heavy metal exposure from the ‘silver’ coatings of recharge cards of mobile phones, the Daily Intake of Metals (DIM), and the health risk index (HRI) were estimated according to Sajjad et al. [28] using the following equations:

DIM = *C*_metal_ × *D*_intake_/*B*_Average weight_where *C*_metal_, heavy metals concentration in coatings; *D*_intake_, intake of metals (approximately 10% of the scratch films is retained under and on the nails); *B*_Average weight_, average body mass of an adult which is 65 kg. The health risk index (HRI) was calculated from the Daily Intake of Metals (DIM) and reference oral dose (*R*_f_*D*). The formula: HRI = DIM/*R*_f_*D*, is used for the calculation of HRI.

According to Refs. [4], [28], [9], if the value of HRI is less than 1 (HRI < 1), the health risk to the population is considered acceptable. On the other hand, if the HRI is equal or greater than 1 (HRI ≥ 1) the population is exposed to unacceptable health risk.

**Fig. 1:**
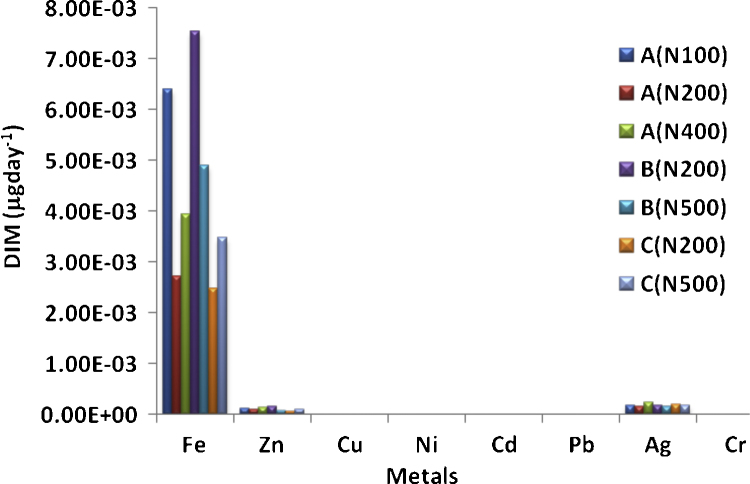
Daily intake of heavy metals in silver coating of the recharge cards.

The estimated DIM values for the metals are presented in Fig. 1. The DIM values are significantly low for all the metals (10^−3^ to 10^−11^ μg day^−1^) and follows the decreasing order Fe > Ag > Zn > Cu > Ni > Pb > Cd > Cr. Iron is the highest, in the magnitude 10^−3^ μg day^−1^, followed by Zn and Ag (10^−3^ μg day^−1^), and Cu (10^−7^ μg day^−1^). Pb, Cd and Cr have very low DIM values in the magnitudes 10^−10^, 10^−10^ and 10^−11^ μg day^−1^ respectively. Going by the serious health concern associated with the intake of Pd, Cd and Cr, the DIM values obtained for these metals in the present study allays the fear of consumers of recharge cards of their associated health risk (unless accumulated for a long time) since ultra trace concentration of Pd, Cd and Cr could only have their ways into the body.

**Fig. 2:**
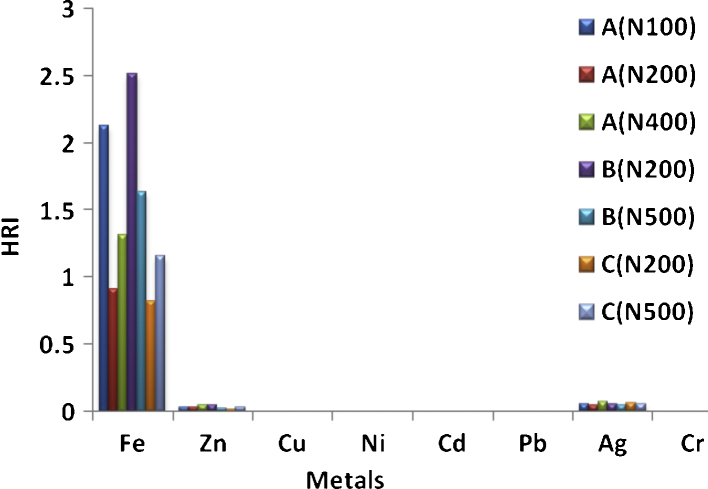
Health risk index of heavy metals.

The health risk index (HRI) has also been recognized as a useful indicator for evaluation of risk associated with the consumption of metals in contaminated food [29]. Therefore, important index was also adapted for the estimation of the health risk associated with the use of recharge cards in this study. The result is presented in Fig. 2. Except for Fe, the HRI of other metals are less than 1 (HRI < 1), which implies that the users of mobile phones recharge cards are exposed to low health risk associated with these metals.

Despite high HRI values obtained for Fe in some brands of the recharge cards, there are few exceptions where low HRI values were recorded (e.g. A  200 (0.907) and C  200 (0.821). The results therefore suggest unregulated and indiscriminate use of iron containing raw materials by the recharge cards makers during production process which leads to variations noticed in this work. Apart from HRI results that demonstrated low health risk associated with these metals, it has also been suggested that the ingested dose of heavy metals is not equal to the absorbed pollutant dose in reality, as a fraction of the ingested heavy metals may be excreted with the remainder accumulated in the body tissues where it affects human health [8].

Table 3, Table 4, Table 5, Table 6 present the results of manganese, copper, cadmium and lead speciation in the recharge card coatings using sequential extraction procedure. This study was carried out to mimic the likely species of the heavy metals in the environment (e.g. soil) when used recharge cards are discharged indiscriminately, or scratched silver coatings are added to environmental dust. This is the common practice by many users of the products. From the results obtained for the different recharge cards denomination studied, it was observed that the metals can exist in the different fractions or species if present in the environment. Table 3 presents Mn species in the coatings. The order of abundance of Mn species in the different fractions is: Mn-oxide > exchangeable > organic > carbonate > residual. Similarly, from Table 4, the order of abundance of Cu species in the different fractions is: Mn-oxide > exchangeable > organic > carbonate > residual.

Table 5, Table 6 present the species distribution of Cd and Pb in the different fractions. For Cd, the order is: Mn-oxide > organic ≥ exchangeable ≥ carbonate > residual; and Pb, Mn-oxide > carbonate ≥ exchangeable ≥vorganic > residual. It is evident from Table 3, Table 4, Table 5, Table 6 that A ( 100) would present the highest abundance form of these metal species when present in the environment since it contains their highest concentration. This result further agreed with the XRF and AAS result where A ( 100) was identified with the higher concentrations of the heavy metals compared with the other brands.

In addition, the predominant form of the metals in the fractions is the Mn-oxide form where Mn, Cu, Cd and Pb had occurred at concentration levels of 992.000, 251.750, 4.250 and 4.875 μg/g respectively. The concentration of Mn in all the individual fractions and in all the samples is highest while concentration of Cd and Pb is lowest. The high association of Mn with the Mn-oxide fraction was attributed to the high oxidation–reduction and cation-exchange reactions. Mn is a very common compound that can be found everywhere on earth. It is one out of three toxic essential trace elements, which means that it is not only necessary for humans to survive but it is toxic when too high concentrations are present in a human body. When people do not take up to the recommended daily allowances their health will be compromised. Also, when the intake is too high health problems will occur. Manganese effects are mainly noticeable in the respiratory tract and in the brain. Symptoms of manganese poisoning are hallucinations, forgetfulness and nerve damage.

The implication of the information presented in Table 3, Table 4, Table 5, Table 6 is that heavy metals present in the silver coatings of recharge cards have the tendency to exist in the different species when present in the environment. These species would enhance their mobility, availability and bioavailablity through the ecosystems, plant soil and water bodies and thereby endangering human life and animals. It is therefore important that the buyer and the users of recharge cards should be careful about indiscriminate disposals of used recharge cards to prevent our environment from heavy metals pollution.

**Fig. 3:**
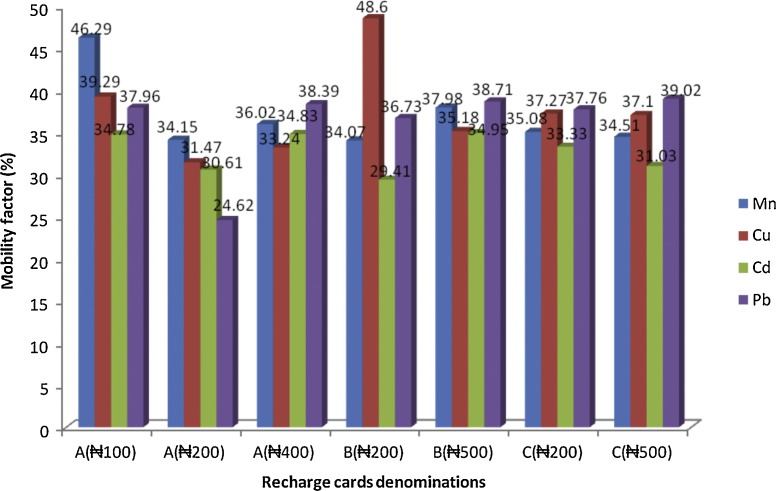
Mobility factors of the metals when present in the environment (values in figure represent the corresponding standard deviation data for the recharge card class).

The values of MF expressed in percentages are presented in Fig. 3. All the metals have reasonably high mobility potential (MF > 30%) through the soil or underground water when present in the environment thus endangering human and animal life. However, this study identified Mn as the most mobile element, which is considered a more environmentally friendly metal compared to the health risks associated with the presence of such metals as Pb and Cd in the environment.

The coefficient of variation (CV), as a statistical tool, can be used to explain the distribution patterns of certain parameters in a given matrix. The higher the CV values, the greater the uneven distribution of the parameter under consideration. Thus, as could be seen in Table 1, the CV values of the elements profiled by XRF ranged between 60.37 in Sc and 236.65 in K. With a CV of not less than 60 across the various recharge cards denominations, it is obvious that the amount of the elements contained in the recharge card coatings probably did not follow a readily determinable pattern. Although the CV values for some of the elements determined by AAS in Table 2 were not as high as those determined by XRF, the pattern remained practically erratic, having a CV values that ranged from 6.65 in Ni to 21.83 in Pb. For the speciation of metals in Table 3, Table 4, Table 5, Table 6, the CV values were highest for the exchangeable fractions. This implies that the greatest uneven distribution of the elements determined was associated with the exchangeable fraction. In the same vein, the residual fraction contained the highest amount of evenly distributed elements.

## 4 Conclusion

It was shown from this study that silver coatings of major recharge cards commonly sold in Nigeria contained high levels of heavy metals such as Fe, Ni, Mn, Cu, Cd, Pb, Cr and Zn. Therefore users of recharge cards samples from A, B and C are potentially exposed to health risks due to heavy metals concentration. Recharge cards A ( 400), C ( 200) and C ( 500) have been considered to be relatively safe for sellers and buyers of the products based on their low metal concentration compared with A ( 100) the use of which should be discouraged in its present form due to the high concentration of heavy metals in its coating. However, despite the high concentration of the metals in the coating, the health risk index (HRI) indicated that the users of mobile phones recharge cards could only experience low health risk associated with these metals. Long term exposure could however be a source of worry.

The results of speciation studies using Mn, Cu, Cd and Pb also confirmed that heavy metals in the recharge card coating are capable of existing in various species including Mn-oxide, carbonate, exchangeable, organic and residual bound forms when present in the environment, with the Mn-oxide species of the metals being the most predominant. Mn and Cu concentrations were very high in all the species compared with Pb and Cd therefore reducing the health risks associated with Pb and Cd toxicity in the environment. The mobility factors estimated for Mn, Cu, Cd and Pb are greater than 30% with Mn identified as the metal with the highest potential to freely move through the soil and percolate underground water when present in the environment. In order to avoid food contamination, proper washing of hands after scratching off coatings on recharge cards should be regularly practiced. Using the printed numbers to replace the mobile phone account should be considered and encouraged since this, apart from being economical, does not readily present metal exposure hazard.

## Conflict of interest

The authors declare that there are no conflicts of interest.
